# Practical Remarks on Fœtal Auscultation

**Published:** 1860-07

**Authors:** R. Druitt

**Affiliations:** L. R. C. P., London


### PRACTICAL REMARKS ON FŒTAL AUSCULTATION.

BY R. DRUITT, L. R. C. P., LONDON.

The readers of the *Medical Times* have been startled by the announcement, on the part of one of the most learned physicians in the world, that the fœtal heart cannot be heard before birth; that if certain sounds be heard, said to be those of the fœtal heart, there is no certainty attainable that they are what they are supposed to be; and that the commonly described sounds are illusions, and exist only in the minds of the listeners. First of all, as to the facts asserted, and the reasonableness of them. To many physicians it is absolutely certain, that at most times, in women pregnant of a live child, especially after the fifth mouth, there can be heard, over some part of the enlarged uterus, a small and distant, but often remarkably distinct heart-beat, varying from 140 to 180 in the minute. By a heart-beat is meant a double beat, of one louder and more pronounced, and another shorter, immediately succeeding the less pronounced.

It is said to be incredible that the sounds of the fœtal heart can reach the ear through so great a mass, consisting of uterine tissues, vessel, and the limbs of the child. But the thickness of the uterus and abdominal walls is not great, and not to be compared in sound deadneing qualities to a common pillow. But who doubts that the ticking of a watch can be heard under a pillow? The writer has heard is own watch through the thickness of sixteen folds of blanket. There is, also, no real difficulty concerning the counting of so large a number of pulsations as 160 in a minute. The breathing of dying children may be distinguished at 180; the pulse at 240. The ticks of a lever watch are five to a second, and may most easily be counted at 240 per minute. When a piano is played rapidly the ear can readily recognize 720 sounds in a minute.

There is no doubt that a woman may be pregnant of a living child, and yet that at times the fœtal heart-sounds not be discovered even by one accustomed to the search. Hence, the absence of the sounds can never be taken as a proof that the fœtus is dead, or that pregnancy does not exist.

Again, sounds may be feebly heard, so that the observer cannot say that they are not heart-sounds; or other sounds may be mistaken for those of the fœtal heart. If the sounds are not confined to a small circle, or are synchronous with those of the maternal heart, they should not be suspected to be fœtal.

Of all the signs which distinguish the enlarged uterus from other tumors, none are more valuable than the following: the uterus, like other hollow viscera, has a regular peristaltic motion, continuous throughout pregnancy, and after delivery, and consisting in periodic contractions, which cause a moderate but decided tension of the organ, and are followed by flaccidity and repose. No other tumor, not tympanitic, can do this; and, during the fits of contraction, the shape, dimensions, and outline of the organ are unmistakable. When about to auscultate, gently shampoo or roll the abdominal parieties over the womb, till it becomes hard and resisting. This is the moment for auscultation. Put the stethescope on the womb, and perpendicular to its surface. Search carefully on the horizontal line on a level with the anterior-superior spine of the ilium; beginning on the left side, then a little above and below; if unsuccessful, go to the right side. Take care that the attidude is easy, and produces no rushing sound in the ears.—*Medical Times and Gazette.*

Poisoning by Arsenic.—Dr. Blondlot has communicated, in a paper, to the Paris Academy of Sciences, a fact, which may be highly valuable in cases of poisoning by arsenic. After numerous experiments, he has come to the conclusion that the slightest quantity of greasy matter in contact with arsenious acid will reduce its solubility to about one-twentieth of what it was before. This explains at once why, in certain judicial investigations, arsenic has been sought for in vain in the liquid portion of the food contained in the stomach, when the food partly consisted of fatty substances, such as broth, milk, &c. It likewise explains how arsenious acid, taken in powder, may sometimes have sojourned a long time in the stomach before it produced any deleterlous effect, since in such cases its action was hindered by the presence of fatty substances. Jugglers have been seen swallowing arsenic with impunity, because, according to Dr. Blondlot, they have previously taken the precaution to drink milk and eat fat bacon. Hence it follows that in cases of poisoning by arsenic, fatty substance may be administered as real antidotes, capable of suspending the action of the poison for a considerable time, until more radical means of effecting a cure can be applied.—*Medical Times and Gazette.*

Losses Sustained by Armies.—M. Meyne, a surgeon in a Belgian regiment of artillery, in a recent work on medical statistics, makes some interesting statements upon this point. He says that an army of 100,000 men, by the sole fact of having entered on a campaign, *i. e.*, leaving out the influence of epidemics and battles, will have 10,000 men in hospital. At the end of some months, if there has been engagements, and the number of patients increase, as is usual, we must count upon a third being placed out of service by disease. During the first fifteen years of the occupation of the Algeria by the French, one-eleventh part of the force were carried off by disease, and a 265th part only by casualties of war, *i. e.*, 23 times as many. Of the 115,000 Russian soldiers who invaded Turkey in 1828–9, but from 10,000 to 15,000 repassed the Pruth, the rest succumbing to fever, dysentery, and pestilence. During the Peninsular wars, of 25,000 French, 3,000 perished on the road from Bayonne to Lisbon either from fatigue or the scorching sun of 1808. The English army, during a period of forty-one months, of an effective force of 61,500 combatants, lost 21,930 by disease, and only 8,889 by the casualties of war. The losses of the French during the Crimean war were 16,000 deaths by the accidents of war, and 53,000 by disease, *i. e.*, 16 to 53; and the proportions were much the same for the Sardinians and the English.

Medical Students in Paris.—The number of inscriptions taken at the Faculty of Medicine of Paris, at the commencement of the present scholastic year, was 988; namely: 922 for the degree of Doctor of Medicine, and 66 for the grade of Officer of Health. Among these are 304 new inscriptions. In 1858, the total number of inscriptions was 1065; the number of new ones, 251. Last year, 34 foreign students were inscribed in the separate register; this year their number is 48.—*Med. News and Library.*

Atropin in Epilepsy.—Dr. Maresch, physician to a lunatic asylum in Virginia, states in the *Medical Times and Gazette* that he succeeded admirably in the treatment of epilepsy with atropin. Of eight females three were cured, and the others greatly improved. Other statistics equally favorable are given. One-fifteenth of a grain of this remedy produces dryness of the throat, difficulty of speech and aberration of vision.

